# Supplementary material for: Element concentrations in pelagic Sargassum along the Mexican Caribbean coast in 2018-2019
Source: PeerJ. 2020 Feb 26;8:e8667. doi: 10.7717/peerj.8667 (PMC7049257; doi:10.7717/peerj.8667)
Supplement: Supplemental Information 1 [file peerj-08-8667-s001.docx]

**Supplementary tables of manuscript:**

**Element concentrations in pelagic *Sargassum* along the Mexican Caribbean coast**

Rodríguez-Martínez, R.E.^1*^, Roy, P.D.^2*^, Torrescano-Valle N.^3^, Cabanillas-Terán, N.^3,4^, Carrillo-Domínguez, S.^5^, Collado-Vides, L.^6^, García-Sánchez, M.^1,7^, van Tussenbroek, B.I.^1^

Supplementary Table 1. Limits of Detection (LOD) of the analyzed elements in Niton FXL energy dispersive XRF (ppm=mg/kg).

| **Element** | **LOD** | **Element** | **LOD** | **Element** | **LOD** | **Element** | **LOD** |
| --- | --- | --- | --- | --- | --- | --- | --- |
| Al | 140 | Cr | 8 | Ni | 10 | Th | 1 |
| As | 4 | Cu | 6 | P | 145 | Ti | 29 |
| Ba | 36 | Fe | 3 | Pb | 2 | U | 4 |
| Ca | 394 | K | 333 | Rb | 1 | V | 3 |
| Cd | 2 | Mg | 2915 | S | 199 | Y | 1 |
| Cl | 266 | Mn | 13 | Si | 342 | Zn | 5 |
| Co | 11 | Mo | 1 | Sr | 6 | Zr | 2 |

Supplementary Table 2. Toxic metals and trace elements maximum levels permitted by different countries in agricultural soils (ppm = mg kg^-1^). nr: no reported.

| **Country** | **Element** | | | | | | |
| --- | --- | --- | --- | --- | --- | --- | --- |
|  | **As** | **Cd** | **Cr** | **Cu** | **Mo** | **Pb** | **Zn** |
| Austria | 50^a^ | 5^a^ | 100^b^ | 100 ^b^ | 10^a^ | 100^b^ | 300^b^ |
| Britain | 20^a^ | 1^a^ | 50 ^b^ | 100 ^b^ | nr | 100^b^ | 300^b^ |
| Canada | 25^a^ | 8^a^ | 75^b^ | 100 ^b^ | 2^a^ | 200^b^ | 400^b^ |
| European Union | nr | nr | nr | 140^b^ | nr | 300^b^ | 300^b^ |
| Germany | 40^a^ | 2^a^ | 200 ^b^ | 200 ^b^ | nr | 1000^b^ | 600^b^ |
| Japan | 15^a^ | nr | nr | 125 ^b^ | nr | 400^b^ | 250^b^ |
| Mexico | 22 ^c^ | 37 ^c^ | 280 ^c^ | nr | nr | 400^c^ | nr |
| Poland | 30^a^ | 3^a^ | 100^b^ | 100 ^b^ | 10^a^ | 100^b^ | 300^b^ |

^a^ Galán and Romero, 2008; ^b^Belmonte et al. 2010; ^c^ NOM-147-SEMARNAT-SSA1-2004
